# Nonalcoholic Fatty Liver Disease and Fibrosis Risk in Patients With Obstructive Sleep Apnea: A Retrospective Analysis

**DOI:** 10.7759/cureus.13623

**Published:** 2021-02-28

**Authors:** Hani A Jawa, Hazim Khatib, Naif Alzahrani, Ahmed Alawi, Mohanad AL-Gamdi, Ahmed Abuljadayel, Sarah Altayyari, Faris Alhejaili, Mahmoud Mosli, Siraj O Wali

**Affiliations:** 1 Department of Medicine, King Abdulaziz University, Jeddah, SAU

**Keywords:** fatty liver disease, obstructive sleep apnoea, liver fibrosis

## Abstract

Objectives

Obstructive sleep apnea (OSA) and nonalcoholic fatty liver disease (NAFLD) are prevalent and commonly associated conditions. We aimed to estimate the prevalence of NAFLD and identify liver fibrosis risk using noninvasive scoring methods in a cohort of patients with OSA.

Methodology

In this retrospective study of patients with confirmed OSA, patients who underwent abdominal ultrasonography were recruited. The primary outcome was the prevalence of suspected NAFLD (steatosis on ultrasound and/or elevated alanine transaminase [ALT]). The secondary outcomes included the prevalence and predictors of liver fibrosis risk as assessed by the NAFLD fibrosis score (NFS) and fibrosis-4 (FIB-4) score.

Results

A total of 133 patients fulfilled the study inclusion criteria. The average age was 49.8±15.1 years, and 57.1% were females. The average BMI was 37.3±14.5. According to the apnea-hypopnea index (AHI) scores, 37.9%, 29.6%, and 32.6% of participants had mild (5-14), moderate (15-29), and severe (>=30) OSA, respectively. Radiologically defined NAFLD was detected in 44.4% of the participants, and elevated liver enzymes were detected in 63.9% of the patients. High NFS and FIB-4 scores were recorded in 9% and 3.8% of the patients, respectively. According to logistic regression analysis, age and BMI significantly predicted high NFS scores.

Conclusion

NAFLD appears to be common among OSA patients. Age and obesity, but not OSA severity, predicted high liver fibrosis risk as assessed by noninvasive scoring systems.

## Introduction

Obstructive sleep apnea (OSA) and nonalcoholic fatty liver disease (NAFLD) are highly prevalent medical conditions, and both are commonly associated with obesity and metabolic syndrome. Globally, the prevalence of NAFLD is estimated to be approximately 25%, whereas the prevalence of OSA in Saudi Arabia is 8.5% (12.8% in males and 4.8% in females) [1-3]. Over the past few years, accumulating evidence has shown OSA to be independently related to the development and progression of NAFLD [4-7]. The mechanisms behind this association are not well identified, but chronic intermittent hypoxia (CIH), as quantified by oxygen saturation, has been shown to be an important trigger [8]. In fact, one study demonstrated that in the absence of obesity, CIH leads to mild liver injury via oxidative stress and excessive glycogen accumulation in hepatocytes, thus sensitizing the liver to a second insult [9].

Liver fibrosis is an established independent predictor of disease-specific and overall mortality in patients with NAFLD [10]. Therefore, early identification and staging of fibrosis have become increasingly important, especially in patients with risk factors for progression from simple steatosis to advanced fibrosis and cirrhosis [11]. Histopathological examination of a liver biopsy is considered the gold standard for staging liver fibrosis [12]. However, it is invasive in nature and carries a small yet potentially serious risk of complications and therefore should be performed only when the expected benefits outweigh the risks, especially in the context of a highly prevalent condition such as NAFLD. Therefore, simple and noninvasive testing using clinical decision aids has been developed and utilized to predict fibrosis risk in patients with NAFLD. Of these tests, two of the most validated are the NAFLD fibrosis score (NFS) and fibrosis-4 (FIB-4) [12]. These scores are calculated using readily available patient demographic data and biochemical tests and can detect advanced fibrosis with modest accuracy when used alone and can reliably exclude advanced fibrosis in a high proportion of patients with NAFLD [11].

The aim of this study was to estimate the prevalence of suspected NAFLD and perform risk stratification of fibrosis among OSA patients using noninvasive scores. Additionally, we aimed to identify possible predictors of liver steatosis and fibrosis in this patient population.

## Materials and methods

We performed a retrospective study of consecutive adult patients evaluated at a Sleep Medicine and Research Center between January 2016 and August 2018. We included all patients with confirmed OSA based on full polysomnography who underwent abdominal ultrasonography for various indications. The following data were collected: (A) Demographics including age, sex, height, weight and body mass index; (B) Clinical parameters including history of metabolic risk factors such as diabetes, hypertension and hyperlipidemia; (C) Laboratory data including complete blood counts, liver enzymes, lipid profile, and hemoglobin A1C; (D) Radiological tests including findings of steatosis or cirrhosis seen on abdominal ultrasound (U/S); and (E) Polysomnography data including the apnea-hypopnea index (AHI). Patients with a history of viral hepatitis or chronic liver disease other than fatty liver and patients with any documented alcohol consumption were excluded.

After completing data collection, the FIB-4 index and NFS were calculated using their respective validated equations as follows:

(A) NFS = (-1.675 + 0.037 × age (years) + 0.094 × BMI (kg/m^2^) + 1.13 × IFG/diabetes (yes = 1, no = 0) + 0.99 × AST/ALT ratio - 0.013 × platelets (×10^9^/L) - 0.66 × albumin (g/dl)).

(B) FIB-4 = (age x AST)/(platelets x sqr ALT).

Definitions and outcomes

Suspected NAFLD was defined as liver steatosis on abdominal US and/or elevated liver enzymes in patients with risk factors. Elevated ALT was defined as >30 IU/L for males and >19 IU/L for females. Liver fibrosis risk was categorized into low risk (NFS <=1.455 and FIB-4 <1.30) and high risk (NFS>0.676 and FIB-4>2.67).

The diagnosis of OSA was made based on the American Academy of Sleep Medicine (AASM) diagnostic criteria [13]. Furthermore, OSA severity was categorized as mild (AHI=5-15), moderate (AHI=15-30), and severe (AHI=>30) based on the AASM recommendations.

The primary outcome of the study was the prevalence of suspected NAFLD among patients with OSA. Secondary outcomes included the prevalence of high and low liver fibrosis risk scores, US-diagnosed liver cirrhosis, factors predictive of NAFLD and high NFS and FIB-4 scores.

Statistical analysis

Descriptive statistics were calculated for baseline characteristics; means and standard deviations (SD) are used to describe continuous variables, and frequencies are used to describe categorical variables. Accordingly, a standard Student’s t-test was used to compare means, and chi-square was used to compare frequencies. Logistic regression analysis was used to study associations with binary dependent variables. Odds ratios (ORs) and 95% confidence intervals (CIs) were estimated. STATA 11.2 (StataCorp, College Station, TX, USA) was used in our analysis. Statistical significance was set at 0.05.

Ethical considerations

This study was approved by the Hospital’s Institutional Review Board (IRB) of (Reference No 378-18).

## Results

Baseline characteristics

A total of 133 patients fulfilled the study inclusion criteria (Table 1).

**Table 1:** Baseline characteristics of 133 patients with confirmed OSA. OSA: obstructive sleep apnea; AHI: apnea-hypopnea index; US: ultrasound; NAFLD: nonalcoholic fatty liver disease; ALT: alanine transaminase; FIB-4: fibrosis-4 score; NFS: NAFLD fibrosis score.

Characteristic	Summary statistics	
	NAFLD on US (n = 59)	No NAFLD on US (n = 74)
Demographics		
Mean age (SD), years	49.7 (1.7)	49.8 (1.9)
Mean BMI (SD), kg/m^2^	38.9 (2.4)	36.0 (1.2)
Female sex	35	41
Comorbidities		
Diabetes mellitus	23	20
Dyslipidemia	7	10
Hypertension	23	27
Liver cirrhosis	1	3
OSA severity		
Mild (AHI = 5-15)	21	29
Moderate (AHI = 15-30)	12	27
Severe (AHI => 30)	26	17
Suspected NAFLD		
Elevated ALT	42	43
Liver fibrosis scores		
High FIB-4	1	4
High NFS	5	7
Low FIB-4	54/59	62/74
Low NFS	33	38

The average age was 49.8±15.1, and 57.1% were females; 37.6%, 32.3% and 12.8% had hypertension, diabetes, and dyslipidemia, respectively. The average BMI was 37.3±14.5. The average serum albumin concentration, AST and ALT were 35.4 (±4.1), 26.4 (±39.9), and 39.5 (±74.3), respectively. The average platelets were 291 (±10.6.2). According to AHI scores, 37.9%, 29.6%, and 32.6% had mild (5-14), moderate (15-29), and severe (>=30) OSA, respectively.

Outcomes

Radiologically suspected NAFLD was detected in 44.4% of participants, biochemically suspected NAFLD (elevated ALT liver enzymes) was detected in 63.9% of participants, and features consistent with liver cirrhosis were found in 3% of this cohort. High NFS and FIB-4 scores were recorded in 9% and 3.8% of all OSA patients, respectively. Low NFS and FIB-4 scores were recorded in 53% and 87% of all OSA patients, respectively.

Predictors of fibrosis

According to logistic regression analysis, age (OR=1.1, p=0.018), liver cirrhosis (OR=1326, p=0.008), and BMI (OR=1.2, p=0.009) significantly predicted high NFS scores, and liver cirrhosis (OR=62.5, p=0.047) predicted high FIB-4 scores (Table 2).

 

**Table 2:** Predictors of high fibrosis risk as assessed by noninvasive scores. BMI: body mass index; AHI: apnea-hypopnea index; ALT: alanine transaminase; FIB-4: fibrosis-4 score; NFS: NAFLD fibrosis score.

Hight NFS	Odds ratio	P	95% confidence interval	95% confidence interval
Age	1.124764	0.018	1.019999	1.240288
Sex	1.190144	0.879	0.1276972	11.09219
AHI severity	1.024474	0.186	0.9883832	1.061882
BMI	1.163037	0.009	1.0382	1.302885
Liver cirrhosis	1326.183	0.008	6.335305	277612.7	
Elevated ALT	0.8622967	0.895	0.0958865	7.754543	
Diabetes	2.269779	0.459	0.2598809	19.82407	
Dyslipidemia	0.2402186	0.456	0.0056482	10.21647	
Hypertension	0.5578992	0.615	0.0573876	5.42367	
High FIB-4	Odds ratio	P	95% confidence interval	95% confidence interval	
Age	1.03609	0.449	0.9451864	1.135736	
Sex	0.8935471	0.0923	0.0909076	8.782836	
BMI	0.9789711	0.715	0.8734246	1.097272	
AHI severity	0.9861778	0.584	0.938203	1.036606	
Diabetes	0.0219631	0.025	0.000781	0.617656	
Dyslipidemia	10.01055	0.177	0.3530267	283.8625	
Hypertension	2.642495	0.419	0.2498476	27.94816	

However, no significant predictors of radiologically suspected fatty liver could be identified statistically (Table 3).

**Table 3:** Predictors of steatosis as assessed by abdominal US. ALT: alanine transaminase; US: ultrasound; AHI: apnea-hypopnea index; BMI: body mass index.

Fatty liver	Odds ratio	P	95% confidence Interval	95% confidence interval
Age	1.000331	0.981	0.9729921	1.028438
Sex	1.561487	0.275	0.7010942	3.477764
AHI	1.01511	0.057	0.9995392	1.030924
BMI	1.014762	0.275	0.9883864	1.041841
Diabetes	2.25053	1.71	0.887361	5.707806
Dyslipidemia	.4374361	0.200	0.1234384	1.550169
Hypertension	.7770351	0.609	0.2951852	2.04544
Elevated ALT	1.560633	0.277	0.7000332	3.479228

## Discussion

In this retrospective study, 133 patients had confirmed OSA and underwent abdominal ultrasonography. Radiologically defined NAFLD was detected in 44.4% of patients, and elevated liver enzymes were detected in 63.9% of patients. High NFS and FIB-4 scores were recorded in 9% and 3.8% of patients with OSA, respectively. Age and BMI were found to significantly predict high NFS scores. Since the establishment of liver fibrosis as a strong predictor of liver-related outcomes and mortality in patients with NAFLD, there has been increasing interest in the development and application of noninvasive, readily available and cost-efficient tests to detect liver fibrosis in patients with this highly prevalent medical condition. Of these tests, FIB-4 and NFS have been recommended for use by practice guidelines for the initial evaluation of patients with NAFLD, especially those at higher risk of nonalcoholic steatohepatitis (NASH), which is the progressive form of NAFLD that can lead to fibrosis and cirrhosis in a subset of patients [11,14]. Patients who are considered to be at a higher risk for NASH include those with advanced age, type 2 diabetes and other components of the metabolic syndrome [12]. No current recommendations, however, are in place regarding whether to routinely screen OSA patients for NAFLD and fibrosis irrespective of the abovementioned NASH risk factors. A recent meta-analysis of 9 studies that included 2272 participants with OSA who underwent bariatric surgery and liver biopsy found OSA and its degree of severity to be independently linked to ALT levels and found OSA to correlate with histological features of NAFLD including fibrosis but not with the NAFLD activity score [6].

The proven association between OSA and NAFLD, irrespective of other risk factors, can be an argument for NAFLD and fibrosis screening in this patient population, especially those with severe sleep apnea. In our study, among 133 patients, most were obese, at least one-third of them had a component of metabolic syndrome, and suspicion of NAFLD was common. Ultrasound-detected steatosis was found in 44.4% of patients. This estimate is considered higher than the prevalence of fatty liver in the general population (approximately 25%) and comparable to that in studies reporting liver biopsy data from bariatric surgery patients with OSA in which steatosis with or without inflammation was found in 44 to 84% of cases [7]. Notably, the sensitivity of US for diagnosing fatty liver is reduced when there is < 30% steatosis and in patients with morbid obesity, which is common in OSA patients [15].

Additionally, we identified 63.9% of cases with elevated ALT using the revisited sex-specific cutoff levels, which may be more sensitive in detecting NAFLD [16]. This is comparable to a recent large French study that assessed the association between OSA and blood markers of liver injury; the study reported elevated ALT in 53% of patients with severe OSA. With regard to the secondary outcomes of this study, the prevalence of low fibrosis risk assessment was different between the two applied scores: 53% using NFS and 87% using FIB-4, which may be due to differences in test performance or the effect of other variables such as patient age. The remaining patients with intermediate or high fibrosis risk would require referral to a hepatology clinic for further assessment by an additional noninvasive tool, namely, elastography or a liver biopsy.

Regarding predictors of fibrosis, we could identify only age and BMI as predictors of high NFS. Both factors are linked to the risk of NASH and fibrosis in NAFLD patients. Expectedly, the finding of liver cirrhosis on US was related to high NFS and FIB-4 scores. With regard to OSA severity, a predictive role for high fibrosis scores was not observed in our cohort, and the results of this association conflict with those of other larger studies [7,17].

Although this study was limited by its retrospective nature, sample size and lack of liver biopsy data, it highlights the magnitude of the prevalence of NAFLD among OSA patients and demonstrates the applicability of NFS and FIB-4 scores as first-line tools for fibrosis risk assessment.

## Conclusions

NAFLD commonly occurs in patients with OSA. Application of the NFS and FIB-4 tools to assess liver fibrosis risk would circumvent the need for liver biopsy in many patients who require careful monitoring and appropriate management, including weight loss, treating metabolic risk factors and continuous positive airway pressure (CPAP) treatment when indicated.
